# Development of a multilocus sequence typing tool for *Balantioides coli* and its genetic diversity in pigs

**DOI:** 10.1128/jcm.00004-26

**Published:** 2026-03-30

**Authors:** Lizhuo Zhao, Ahui Xu, Kai He, Zhenjie Zhang, Weifeng Qian, Min Zhang, Zhiguo Wei, Tianqi Wang, Suhui Hu, Wenchao Yan

**Affiliations:** 1 Parasitology Laboratory, College of Animal Science and Technology, Henan University of Science and Technology 74623, Luoyang, China; 2 College of Animal Science and Technology, Tarim University 12483, Alar, China; Mayo Clinic Minnesota, Rochester, Minnesota, USA

**Keywords:** pigs, *Balantioides coli*, MLST, microsatellite, genetic polymorphism

## Abstract

*Balantioides coli* is a globally significant zoonotic protozoan, yet effective genotyping tools such as multilocus sequence typing (MLST) remain limited for this pathogen. In this study, we screened microsatellite sequences from the whole-genome data of a pig-derived *B. coli* isolate (P011) and evaluated 12 candidate loci (BC-MS1–BC-MS12) using 45 porcine isolates. Five loci (BC-MS1, BC-MS5, BC-MS10, BC-MS11, and BC-MS12), which demonstrated high amplification efficiency and polymorphism, were selected to develop an MLST tool. Sequence analysis revealed 14–20 subtypes per locus, and 22 MLS subtypes were identified among the 29 isolates successfully sequenced at all five loci. The BC-MS5 locus is located in the encoding region and the transcriptional termination regulatory region of the *B. coli* genome, and it is the locus with the greatest variability among the five selected loci. At the BC-MS5 locus, 42 isolates were classified into five groups, with group 5 and group 1 being the dominant populations across the six provinces. No obvious geographical isolation was observed among the pig-derived *B. coli* isolates. This is the first development of a high-resolution MLST tool for *B. coli*, offering new insights into its genetic diversity and transmission dynamics.

**IMPORTANCE:**

The 18S rDNA and ITS1-5.8S rDNA-ITS2 genetic markers exhibit some limitations, including low resolution in the molecular epidemiological study of *Balantioides coli*. Here, five microsatellite loci (BC-MS1, BC-MS5, BC-MS10, BC-MS11, and BC-MS12) with high amplification efficiency and polymorphism were selected from genomic data of *B. coli* to develop a multilocus sequence typing (MLST) method for the first time. Sequence analysis reveals that there are multiple MLS subtypes and groups among pig-derived isolates in China and no obvious geographical isolation. The established MLST method demonstrates higher resolution than conventional genotyping approaches relying on 18S rDNA or ITS1-5.8S rDNA-ITS2 and will serve as a useful tool for investigating the transmission dynamics and genetic diversity of this zoonotic pathogen.

## INTRODUCTION

*Balantioides coli* is an important zoonotic protozoan with a wide host range (1, 2). It commonly causes diarrhea in animals, posing a serious threat to animal husbandry and public health, especially in weaned pigs (3, 4). *B. coli* was first discovered in Sweden in 1857 from fecal samples of two dysentery patients, and Malmsten called it *Paramecium coli* at that time (5, 6). Over the years, there has been considerable controversy about the name and classification of *B. coli*, which is due to insufficient taxonomic and genetic data. It remained unresolved until Pomajbíková et al. demonstrated that *B. coli* isolates from mammals and ostriches exhibit significant genetic differences from the frog-derived *Balantidium entozoon*, which is the type species of the genus *Balantidium* (5). Subsequently, some scholars acknowledged that *Balantioides* has priority as a genus name and revised its taxonomy. The pathogen is formally designated *Balantioides coli*, and the associated clinical disease is termed balantiosis (7–9).

Genotyping or subtyping of isolates plays a crucial role in tracing pathogens, identifying outbreak cases, and making evolutionary pathway and lineage comparisons on a global scale. At present, two molecular diagnostic tools targeting the 18S rDNA and ITS1-5.8S rDNA-ITS2 regions have been used to characterize the transmission of *B. coli* in various hosts (10, 11). However, among these genetic markers, the regions encoding the 18S and 5.8S ribosomal RNAs (rRNAs) are highly conserved and have low resolution for differences among various isolates, which was only suitable for interspecific identification. Although internal transcribed spacer 1/2 (ITS1/2) regions can be used for intraspecific typing, they exhibit high variability, even in the same sample or trophozoite. There were multiple ITS genetic variants that are not strongly related to the biological properties, such as infectivity and pathogenicity of *B. coli* isolates.

Multilocus sequence typing (MLST), a high-resolution typing method, has become an essential tool for studying the molecular epidemiology, subtype analysis, and population genetic structure of many pathogens (12–14). This approach reveals multilocus subtypes by amplifying and sequencing target loci, including housekeeping genes, microsatellites, and minisatellites, and followed by analyzing the relationships among different isolates. Microsatellites are widely used as genetic markers in parasite classification and population genetics research, as demonstrated in studies of *Toxoplasma gondii* (15), *Neospora caninum* (16), *Giardia duodenalis* (17), *Enterocytozoon bieneusi* (18), *Cryptosporidium* spp. (19), and *Cyclospora cayetanensis* (20, 21). Their utility stems from several advantageous characteristics, including good repeatability and fidelity, abundance in genomes, codominant inheritance, and high variability in repeat numbers across individuals (22, 23). However, an MLST tool for *B. coli* has not yet been reported.

Pigs are the primary natural host of *B. coli*, and most reported human cases are also associated with pigs (24). An efficient *in vitro* culture system for *B. coli* based on modified DMEM medium has been established, providing a sufficient number of high-quality trophozoites for whole-genome sequencing of this pathogen. The complete genome sequence of *B. coli* was obtained in previous studies (25, 26). In this work, microsatellite sequences were screened from the *B. coli* genome, and an MLST tool was successfully developed. It will serve as a powerful tool for analyzing genetic diversity, identifying infection sources, tracing spatiotemporal distribution, and investigating outbreaks and endemic characteristics of *B. coli*.

## MATERIALS AND METHODS

### Specimens

A total of 45 *B. coli*-positive DNA samples (Henan, *n* = 20; Shaanxi, *n* = 5; Heilongjiang, *n* = 5; Jiangsu, *n* = 6; Guangdong, *n* = 4; and Hunan, *n* = 5), identified by 18S rRNA gene analysis, were included in this study. Fecal samples from weaned piglets were collected from nine pig farms across six provinces in China (four farms in Henan and one each in Shaanxi, Heilongjiang, Jiangsu, Guangdong, and Hunan). The samples were placed into sterile 50 mL centrifuge tubes, transported on ice, and stored at −80°C until DNA extraction. Genomic DNA was extracted using the E.Z.N.A. Stool DNA Kit (Omega Bio-Tek, Norcross, GA, USA) according to the manufacturer’s instructions.

Among these samples, 8 DNA samples were used for PCR primer evaluation and amplification optimization using a Genesy 96T gene amplification thermal cycler (Tianlong, Xi'an, China), while 45 DNA samples were analyzed at the selected microsatellite loci. Furthermore, ITS genetic variants of the relevant samples were identified by a polymerase chain reaction-restriction fragment length polymorphism typing method established in our laboratory (27).

### Identification of microsatellite

Microsatellite sequences were retrieved from the genomic data of P011 (26), a *B. coli* strain of porcine origin from Henan Province, China. These sequences were identified using the Simple Sequence Repeat Identification Tool (https://archive.gramene.org/db/markers/ssrtool) (28) and further verified through the Tandem Repeats Finder (https://tandem.bu.edu/trf/trf.html) website (29).

### PCR amplification of the microsatellite loci

Based on the sequences flanking the microsatellite repeats, three sets of specific nested PCR primers were designed for each locus using Oligo six software. The PCR reaction mixture had a total volume of 25 μL, containing 12.5 μL of the mix (2× EasyTaq PCR SuperMix [+dye]), 9.5 μL of ddH_2_O, and 1 μL of each primer (25 μM). Notably, 1 μL of DNA template was added in the primary PCR, while 1 μL of the primary PCR product was used as template in the secondary PCR. The PCR protocol consisted of an initial denaturation at 94°C for 5 min; followed by 35 cycles of denaturation at 94°C for 50 s, annealing at the temperatures specified in Table 1 for 50 s, and extension at 72°C for 50 s, with a final extension at 72°C for 10 min. The secondary PCR products were detected by 1.5% agarose gel electrophoresis. Each DNA sample was analyzed by PCR at least twice.

**TABLE 1:** The optimal primers and PCR amplification efficiencies of the selected five microsatellite loci

Locus	Targeted repeat	Primer sequence (5′−3′)	Annealing temp (°C)	Expected size (bp)	Amplification efficiency*^a^* (%)
BC-MS1	(TA)_6_	F_1_: GGCGTATCAGTCTATCACA R_1_: ATCGAAAGACTCTCACACC	52	466	77.6
		F_2_: ACTCTATTTGACTATGAAGG R_2_: TCGACTGTGGATGATAAC	54	380	
BC-MS5	(TA)_5_	F_1_: GTCATTCGAGCATAGTGT R_1_: TCAGTCCAAAGCAGTATC	52	439	72.4
		F_2_: CCATTAACGTATGAAGAACG R_2_: TGGTAAACCTAAACCACC	54	340	
BC-MS10	(TA)_5_	F_1_: CGTGCAATAAAGAGAGATGT R_1_: CAAAGTGATATCAGAGGCA	53	440	82.8
		F_2_: CAGCTAGGGAATTGAACT R_2_: GACAGAGAAATGGTATAAGG	54	352	
BC-MS11	(TCT)_6_	F_1_: GACCTTGAGCTCTGTTTAC R_1_: GATGTCCTAGATAATGCGA	54	404	91.4
		F_2_: CACTTGAGCTCTGTTTAC R_2_: AGAGTGGAAGCTTCAGTG	54	351	
BC-MS12	(ATT)_5_	F_1_: CCAGCATTCTTATACAGTG R_1_: TAGCTATGATGTGGAGTC	53	437	74.1
		F_2_: AAGAGTTATCAGAGGCGA R_2_: TTAGTCTCAGAGGGAAAG	56	350	

^*a*^ No. of PCR positive samples/total No. of samples.

### Sequence analysis

The positive secondary PCR products for each locus were analyzed by 2% agarose gel electrophoresis and then purified using the StarPrep Gel Extraction Kit (GenStar, Beijing, China) following the manufacturer’s instructions. The purified PCR products were subsequently sequenced bidirectionally on an ABI 3730 Genetic Analyzer (Sangon Biotech, Shanghai, China).

The obtained sequences were checked with Chromas 2.6.6 software (https://technelysium.com.au/wp/chromas/). Only sequences with minimal overlapping peaks were considered suitable for subsequent analysis. Consensus sequences were generated by assembling available sequencing results using the SeqMan program in Lasergene 7.1.0 (https://www.dnastar.com/software/lasergene/) (30).

Sequences from each locus were aligned with the P011 reference sequence using the MegAlign program in Lasergene 7.1.0. Variations (including base substitutions, insertions, or deletions) within both the microsatellite repeat region and its flanking sequences were systematically characterized. (Note that variations observed at the same position in fewer than three isolates were excluded as potential artifacts.) Phylogenetic relationships among different *B. coli* isolates were determined by constructing a neighbor-joining tree in MEGA 7 software (31). The diversity of each locus was assessed using DnaSP 5.10.1 (www.ub.es/dnasp/) (32).

### Nucleotide sequence accession numbers

The sequences generated in this study have been deposited in the GenBank database under accession numbers PX047962–PX047981.

## RESULTS

### Initial evaluation of the microsatellite loci

A total of 12 microsatellite loci (BC-MS1–BC-MS12) were initially identified from a scaffold of the whole-genome sequence of *B. coli* strain P011. The amplification conditions and results of the 36 sets of nested PCR primers were preliminarily evaluated using eight *B. coli* DNA samples. Of these, 22 primer pairs were excluded due to non-specific amplification or unreadable sequencing chromatograms, and two primer pairs for BC-MS8 were discarded because no samples were successfully amplified at this locus. After screening, optimal nested PCR primers were selected for each locus, with expected PCR product sizes ranging from 340 to 380 bp (Table 1).

**Fig 1:**
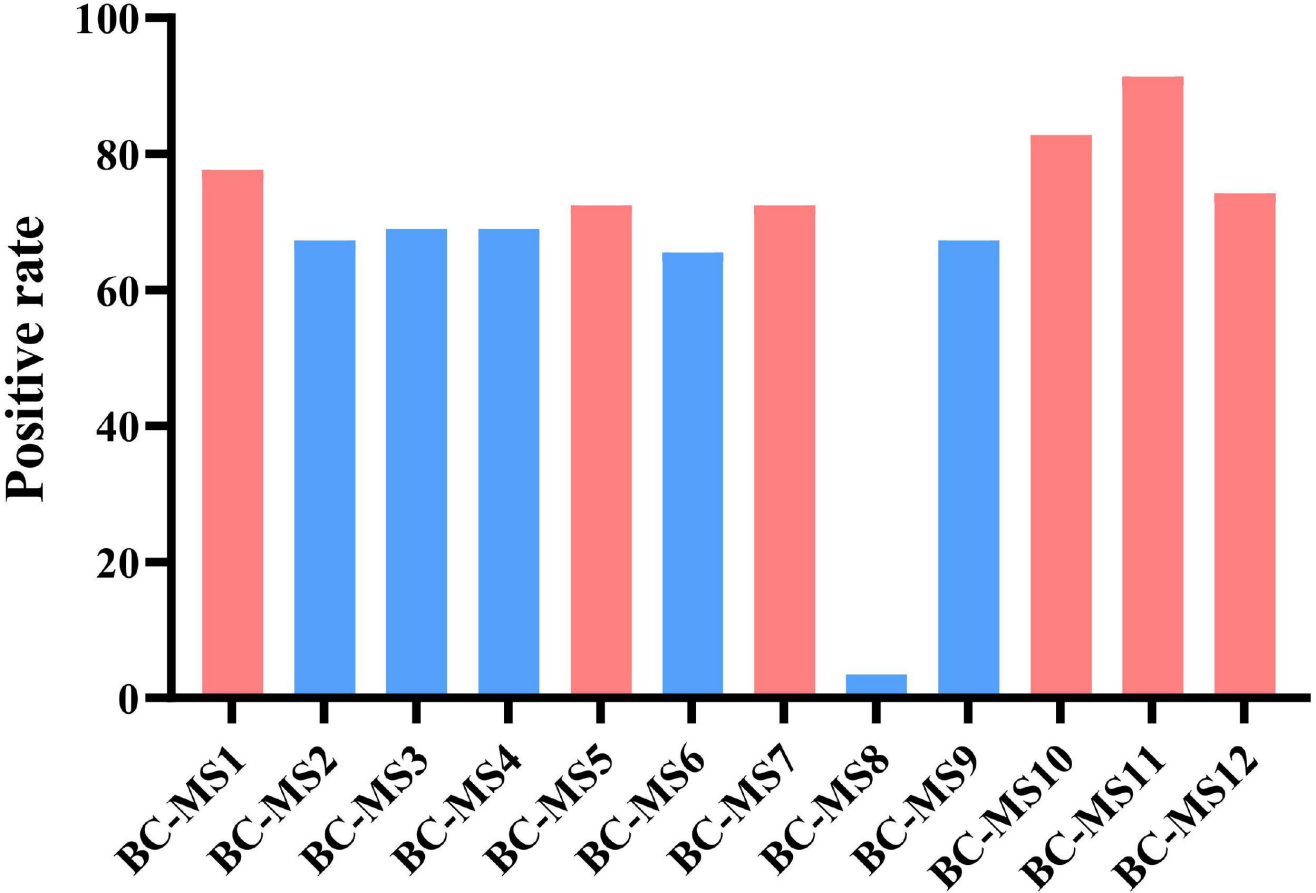
PCR amplification efficiency of 12 microsatellite loci.

The amplification efficiency of the selected primers was further validated using 15 samples. The BC-MS11 locus exhibited the highest amplification efficiency (91.38%), while BC-MS8 showed the lowest (3.45%). Based on these results, the six loci with the highest amplification efficiency—BC-MS1, BC-MS5, BC-MS7, BC-MS10, BC-MS11, and BC-MS12—were selected for subsequent analysis (Fig. 1; Fig. S1). Positive PCR products were purified and subjected to Sanger sequencing. Sequence analysis of a subset of DNA samples at the six loci revealed that both the repeat and flanking regions of BC-MS7 were highly conserved, limiting its usefulness for strain discrimination (Fig. S2). In contrast, while the repeat region of BC-MS5 was also conserved, substantial variation in its flanking sequences enabled reliable discrimination between strains (Fig. S2 and S3). Owing to its high conservation and limited polymorphism, BC-MS7 was ultimately excluded. Consequently, five microsatellite loci (BC-MS1, BC-MS5, BC-MS10, BC-MS11, and BC-MS12), which showed high amplification efficiency and substantial polymorphism, were selected for MLST and genetic diversity analysis in this study.

### Nature of polymorphism in microsatellite sequences

The repeat regions of all five selected loci were located outside the coding regions of the respective sequences. The microsatellite repeats of BC-MS1 and BC-MS5 were situated in the 3′-UTR of their corresponding genes, while those of BC-MS12 were located in the 5′-UTR of its gene. Both BC-MS10 and BC-MS11 sequences originated from non-coding regions or pseudogene sequences in the *B. coli* genome. Hypervariable regions in BC-MS1, BC-MS10, and BC-MS12 were predominantly located in the upstream of the microsatellite fragment, whereas the microsatellite repeat region of BC-MS11 itself exhibited high variability (Table 2).

**TABLE 2:** The nature of the five selected microsatellite loci^*a*^

Locus	Upstream	Microsatellite	Downstream	Hypervariable region
BC-MS1	Coding region	3′-UTR	3′-UTR	Coding region
BC-MS5	Coding region	3′-UTR	3′-UTR	3′-UTR
BC-MS10	Non-coding region	Non-coding region	Non-coding region	Non-coding region
BC-MS11	Non-coding region	Non-coding region	Non-coding region	Non-coding region
BC-MS12	5′-UTR	5′-UTR	Coding region	5′-UTR

^*a*^ The nature of each locus was identified in the genome of *B. coli* P011 strain.

Comparative analysis of repeat units, insertions/deletions (indels), and single-nucleotide polymorphism (SNP) quantity and distribution across sample sequences revealed four variation patterns for each microsatellite locus: (i) variable numbers of repeat units (e.g., BC-MS1 and BC-MS10); (ii) indels within repeat sequences (e.g., BC-MS1, BC-MS10, and BC-MS11); (iii) base mutations in repeat sequences (e.g., BC-MS11 and BC-MS12); and (iv) polymorphisms (indels/substitutions/SNPs) in flanking sequences (Fig. S2). Notably, BC-MS5 showed the highest overall polymorphism, with its hypervariable regions located in the flanking sequence of the repeat region. Genetic polymorphism metrics, such as its highest haplotype diversity (*Hd*), further support this observation (Fig. S3; Table 1). Additionally, when only the microsatellite region was considered, BC-MS11 exhibited greater polymorphism than the other four selected loci. In contrast, BC-MS5 demonstrated higher polymorphism across the entire fragment sequence.

### Genetic relationship among various strains of *B. coli* from pigs based on the microsatellite sequences

**Fig 2:**
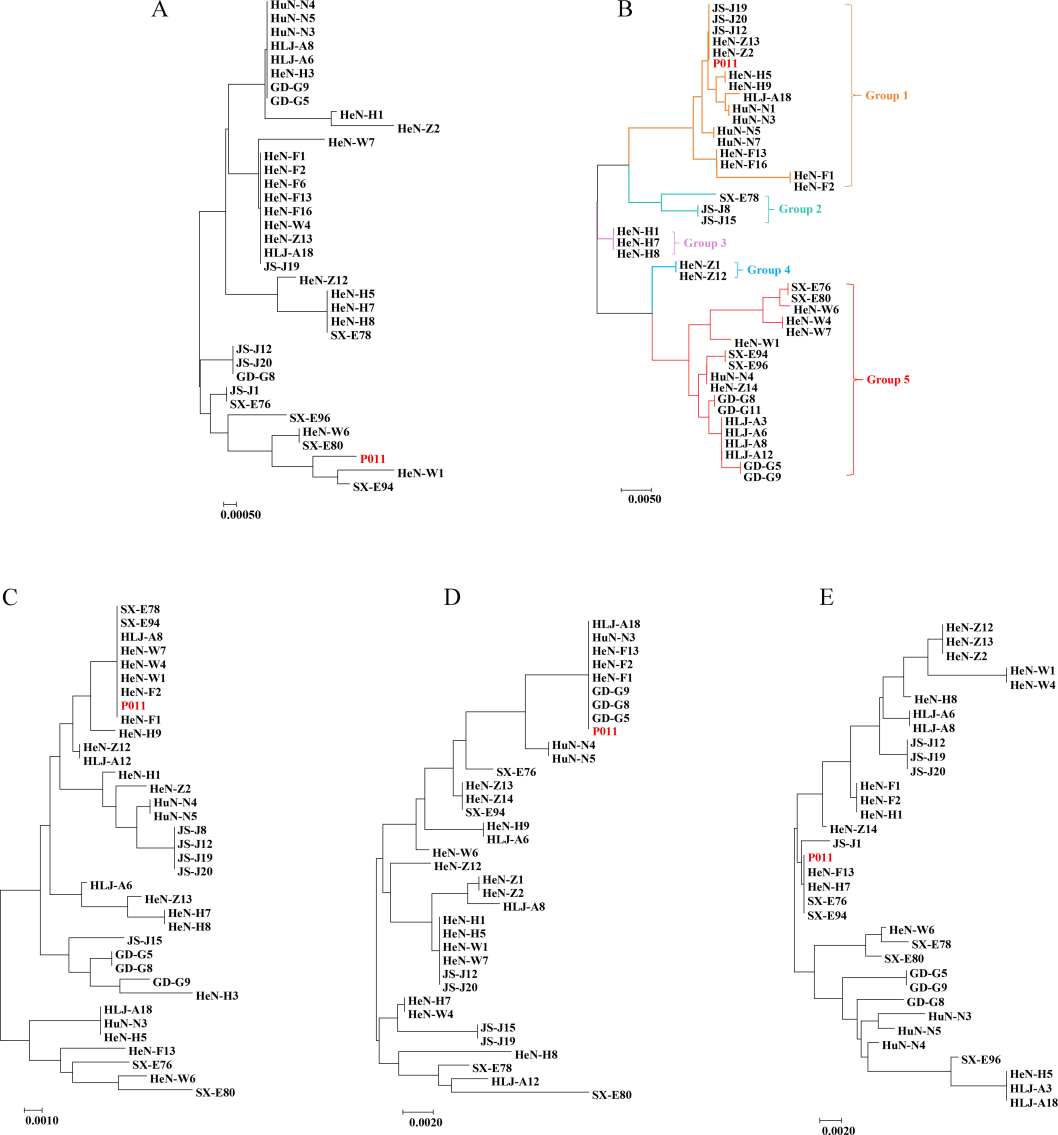
Phylogenetic trees of *B. coli* from pigs at the five microsatellite loci. (**A**) BC-MS1, (**B**) BC-MS5, (**C**) BC-MS10, (**D**) BC-MS11, and (**E**) BC-MS12. GD, Guangdong Province; HeN, Henan Province; HLJ, Heilongjiang Province; HuN, Hunan Province; JS, Jiangsu Province; SX, Shaanxi Province. P011 is the reference strain isolated from a weaned pig in Luoyang, and its whole genome has already been sequenced and assembled.

The sequence polymorphisms of 45 samples from nine pig farms were analyzed using the five microsatellite loci described above. The nucleotide sequences obtained for each locus showed homology with the reference strain P011. Sequence similarities at four loci (BC-MS1: 98.7%–100%, BC-MS10: 98%–100%, BC-MS11: 97.7%–100%, and BC-MS12: 97.4%–100%) were higher than that of BC-MS5 (92.5%–100%), which exhibited the greatest variability among the five loci. Based on variations in the microsatellite repeat regions, the numbers of subtypes at BC-MS1, BC-MS5, BC-MS10, BC-MS11, and BC-MS12 were 2, 1, 3, 6, and 2, respectively. When all variations across the entire sequences—including repeat unit numbers, indels, and SNPs—were considered, the 45 *B. coli* isolates displayed greater genetic diversity, with more subtypes identified per locus (BC-MS1: 14, BC-MS5: 20, BC-MS10: 19, BC-MS11: 16, and BC-MS12: 19) (Table 3; Fig. 2).

**TABLE 3:** The specimens used in this study and the number of their subtypes at the five selected microsatellite loci

Pig farms	Locations	Number of isolates	Number of subtypes				
			BC-MS1	BC-MS5	BC-MS10	BC-MS11	BC-MS12
F	Luoyang, Henan	5	1	3	2	1	2
H	Luoyang, Henan	6	3	2	5	4	4
Z	Luoyang, Henan	5	3	4	3	3	2
W	Jiaozuo, Henan	4	4	3	2	3	2
A	Harbin, Heilongjiang	5	2	2	4	4	2
E	Weinan, Shaanxi	5	5	3	3	4	4
J	Suzhou, Jiangsu	6	3	3	2	2	2
G	Guangzhou, Guangdong	4	2	2	2	1	2
N	Chenzhou, Hunan	5	1	3	2	2	3
Total		45	14	20	19	16	19

Samples sharing a sequence similarity greater than 99% and clustering on the same branch of the phylogenetic tree were classified into one group (33). Phylogenetic analysis of the sequences obtained from 45 isolates revealed that these isolates were distributed across multiple groups at each of the five loci (BC-MS1: six groups, BC-MS5: five groups, BC-MS10: five groups, BC-MS11: seven groups, and BC-MS12: five groups) (Fig. 2). At the BC-MS5 locus, 42.9% and 38.1% of the samples belonged to group 5 and group 1, respectively. Samples assigned to group 1 or group 5 originated from different pig farms and even from different provinces. While most isolates from a single pig farm generally fell into the same group, a small proportion of isolates from the same farm were assigned to a different group. A similar pattern was observed at the other four loci (BC-MS1, BC-MS10, BC-MS11, and BC-MS12). These results suggest that the distribution of *B. coli* does not exhibit obvious geographical isolation (Fig. 2).

### Multilocus sequence subtypes of *B. coli* in pigs

**Fig 3:**
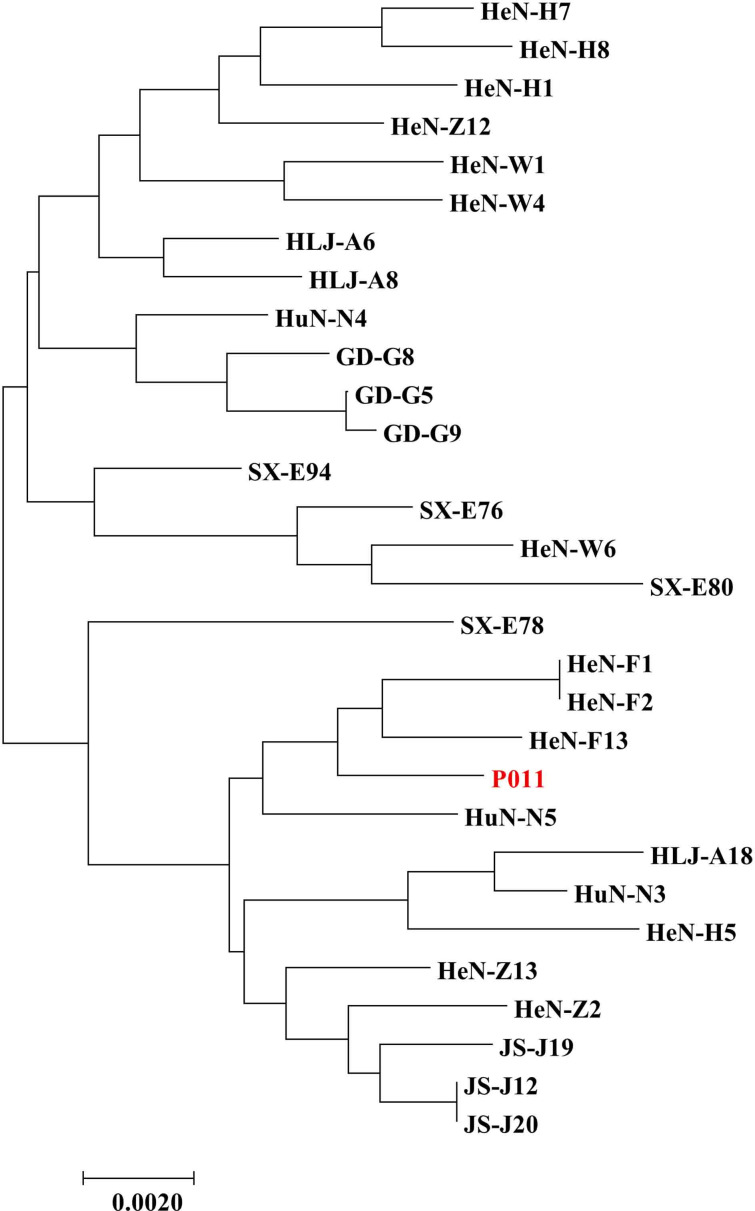
Phylogenetic relationship among *B. coli* isolates based on the concatenated five loci (BC-MS1, BC-MS5, BC-MS10, BC-MS11, and BC-MS12). P011 is the reference strain isolated from a weaned pig in Luoyang.

A total of 29 isolates were successfully amplified and sequenced at all five loci (BC-MS1, BC-MS5, BC-MS10, BC-MS11, and BC-MS12). The sequences from each isolate were concatenated across these loci, resulting in a combined contig of approximately 1,781 bp. A neighbor-joining tree was constructed using the concatenated sequences (Fig. 3). MLST analysis identified 22 subtypes of *B. coli*. Most MLS subtypes contained only one or two isolates, except for three subtypes that each comprised three isolates. The 29 isolates were classified into six groups. Phylogenetic analysis based on the concatenated sequences yielded results consistent with those obtained for BC-MS5 alone, and no clear geographical isolation was observed among the pig-derived *B. coli* isolates. In this study, most isolates were identified as genetic variant B based on the ITS1-5.8S rRNA-ITS2 marker, indicating that this is the dominant genetic variant of *B. coli* in pigs in China, a finding consistent with previous reports (34). Typing results based on the ITS1-5.8S rRNA-ITS2 sequence showed no correlation with those based on the five selected microsatellite loci.

## DISCUSSION

Pathogen genotyping is important for distinguishing circulating subtypes, tracing infection sources during outbreaks, and characterizing subtypes associated with specific clinical presentations. In this study, five microsatellite markers were identified by analyzing the genome sequence of *B. coli*, and a high-resolution MLST tool for *B. coli* was successfully developed. This novel tool revealed substantial genetic polymorphism among clinical *B. coli* isolates, demonstrating superior discriminatory power compared to traditional genotyping targets. For example, genotyping based on the ITS1/2 region—a marker currently widely used for *B. coli*—identified only three genetic variants (A, B, and C) in the studied samples (35). In contrast, MLST captures the full variability within sequenced regions. This approach not only detected significant sequence variations in non-repetitive regions, including deletions, insertions, and SNPs, but also revealed differences in the repeat regions of microsatellite markers among *B. coli* isolates. These findings indicate that the MLST tool based on the five microsatellite loci offers higher resolution than ITS-based genotyping. This tool will aid in accurately discriminating *B. coli* isolates and elucidating their genetic evolutionary relationship.

Microsatellites are widely used as genetic markers in eukaryotes. Owing to their high polymorphism in repeat unit number and their broad distribution across the genome, these markers can be efficiently detected by PCR and are therefore commonly applied in subtype identification, genetic relationship analysis, and population genetic structure studies. All markers identified in this study were characterized as simple sequence repeats. These repeats consisted of five or six motifs of two to three nucleotides and exhibited high A/T content, consistent with features of the *B. coli* genome. Among the analyzed loci, BC-MS5 showed the highest level of polymorphism. Although its microsatellite repeat region was highly conserved, substantial sequence variation was observed in its downstream flanking region. BC-MS11 also served as a highly polymorphic marker, displaying variation in both the repeat region and its flanking sequences across *B. coli* isolates. Furthermore, the microsatellite repeat regions of all five selected loci were located in non-coding regions of the *B. coli* genome. Although non-coding regions do not encode proteins, they may play important roles in gene expression regulation, as they contain promoter and transcription termination sequences (3, 6). Numerous genome-wide association studies have revealed that approximately 93% of common disease-associated genetic variants are located in non-coding regions, likely because non-coding sequences exhibit features such as linkage inheritance, long-distance effects, and strong dynamic regulation (3, 7–39).

The MLST analysis conducted in this study revealed significant genetic diversity among *B. coli* isolates from nine pig farms in China. Among 45 pig-derived isolates, 14– 20 subtypes were identified per locus, and 22 MLS subtypes were observed among the 29 isolates successfully sequenced at all five loci. Notably, BCMS5 exhibited the highest resolution of the five loci, grouping 42 isolates into five groups, with group 1 and group 5 being the dominant populations across the nine farms. Within the same farm, multiple subtypes were present; although most samples belonged to the same group, a small number fell into different groups. These findings suggest that *B. coli* infection in pigs in China shows no clear geographical isolation. This pattern may be attributed to the high prevalence of *B. coli* in pigs, driven by intensive breeding practices and interregional trade. Moreover, frequent transmission likely increases opportunities for mixed infections and genetic recombination among different *B. coli* isolates, while relatively low biosecurity measures on pig farms may further facilitate pathogen introduction (40–42). Additionally, the high genetic diversity observed in this study implies a potential association between genotype and clinical presentation, where different genotypes may lead to varied symptoms. In particular, acute cases of *B. coli* in weaned piglets may be linked to specific genotypes, and the relationship between genotype and virulence warrants further investigation.

In conclusion, we have successfully developed an MLST tool for *B. coli* subtyping, which revealed substantial genetic diversity among pig-derived *B. coli* strains in China. The MLST offers higher resolution than conventional ITS-based identification, allowing multiple MLS subtypes to be distinguished even within the same ITS genetic variant. Furthermore, MLST analysis indicated no clear geographical isolation pattern among the pig-derived *B. coli* isolates. These findings provide new insights into the molecular epidemiology of *B. coli* in Chinese pig farms. Notably, the weaned pigs infected with *B. coli* tend to exhibit more severe clinical symptoms than other hosts, suggesting the existence of possible host-adapted genetic features. However, current knowledge of *B. coli* genetic variation remains limited. Future studies should expand the sample size to include broader geographical regions and diverse host species, which will help elucidate the population genetic structure of this parasite and clarify the relationships between specific genotypes or subtypes and clinical manifestations.

## Data Availability

The raw data supporting the conclusions of this article will be made available by the authors without undue reservation.

## SUPPLEMENTAL MATERIAL

Supplemental figures and tables jcm.00004-26-s0001.pdf Information of samples and microsatellite loci in this paper.

ASM does not own the copyrights to Supplemental Material that may be linked to, or accessed through, an article. The authors have granted ASM a non-exclusive, world-wide license to publish the Supplemental Material files. Please contact the corresponding author directly for reuse.
