# Supplementary material for: Development of a multilocus sequence typing tool for Balantioides coli and its genetic diversity in pigs
Source: J Clin Microbiol. 2026 Mar 30;64(5):e00004-26. doi: 10.1128/jcm.00004-26 (PMC13170333; doi:10.1128/jcm.00004-26)
Supplement: Supplemental figures and tables — Information of samples and microsatellite loci in this paper. [file jcm.00004-26-s0001.pdf]

TABLE S1. Evaluation of genetic polymorphism at each locus

Locus	Isolates	Lenth	*S*	*H*	*Hd*
BC-MS1	36	380	5	14	0.881
BC-MS5	43	340	23	20	0.959
BC-MS10	36	354	9	19	0.922
BC-MS11	36	354	10	16	0.906
BC-MS12	34	350	12	19	0.955

S: Variable (monomorphic) sites; *H*: Number of Haplotypes; *Hd*: Haplotype diversity.

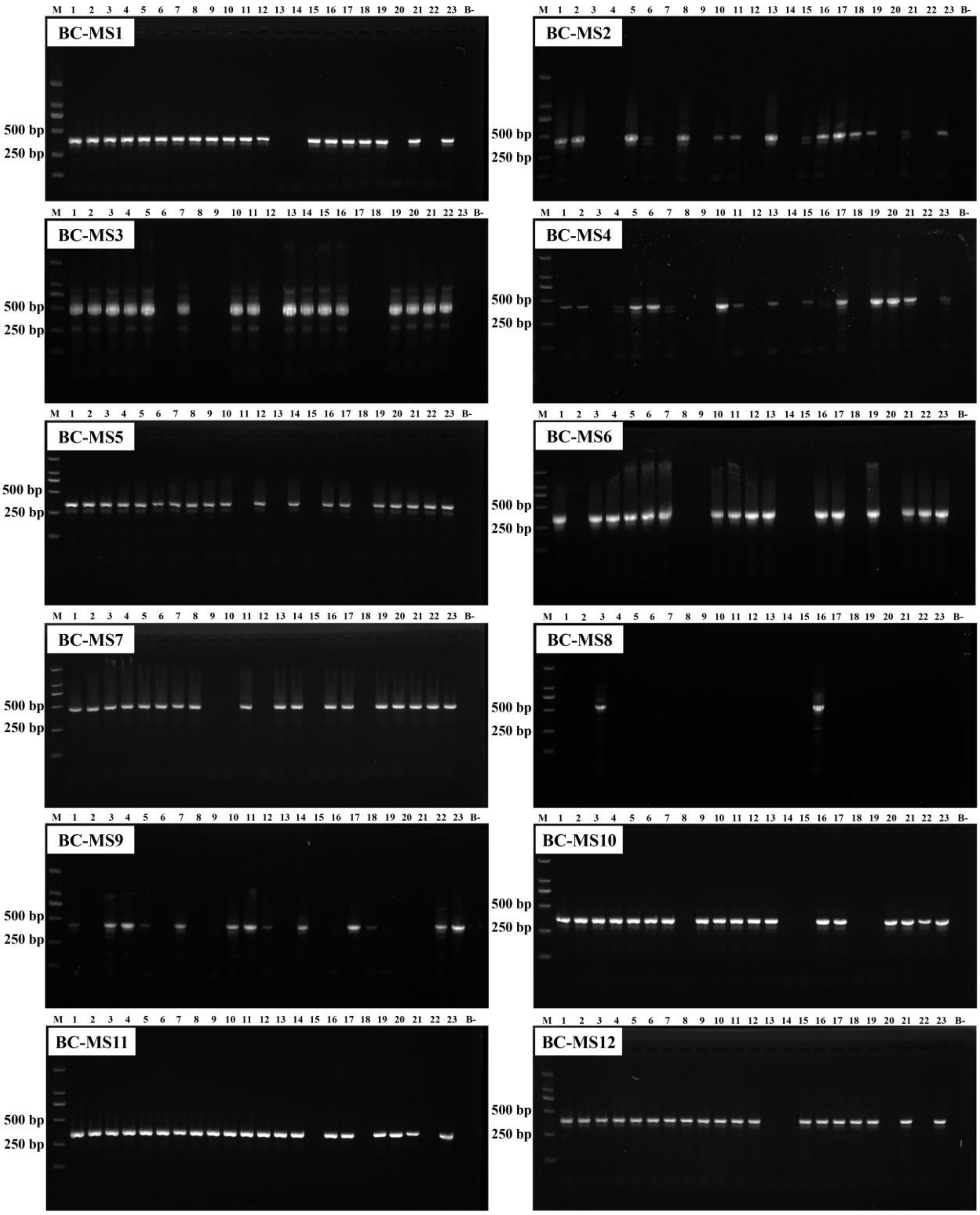

FIG S1. PCR amplification results of partial *B. coli* samples at 12 microsatellite loci. The number on the upper side of each sub-image represents samples, M represents DNA marker, B- is negative control.

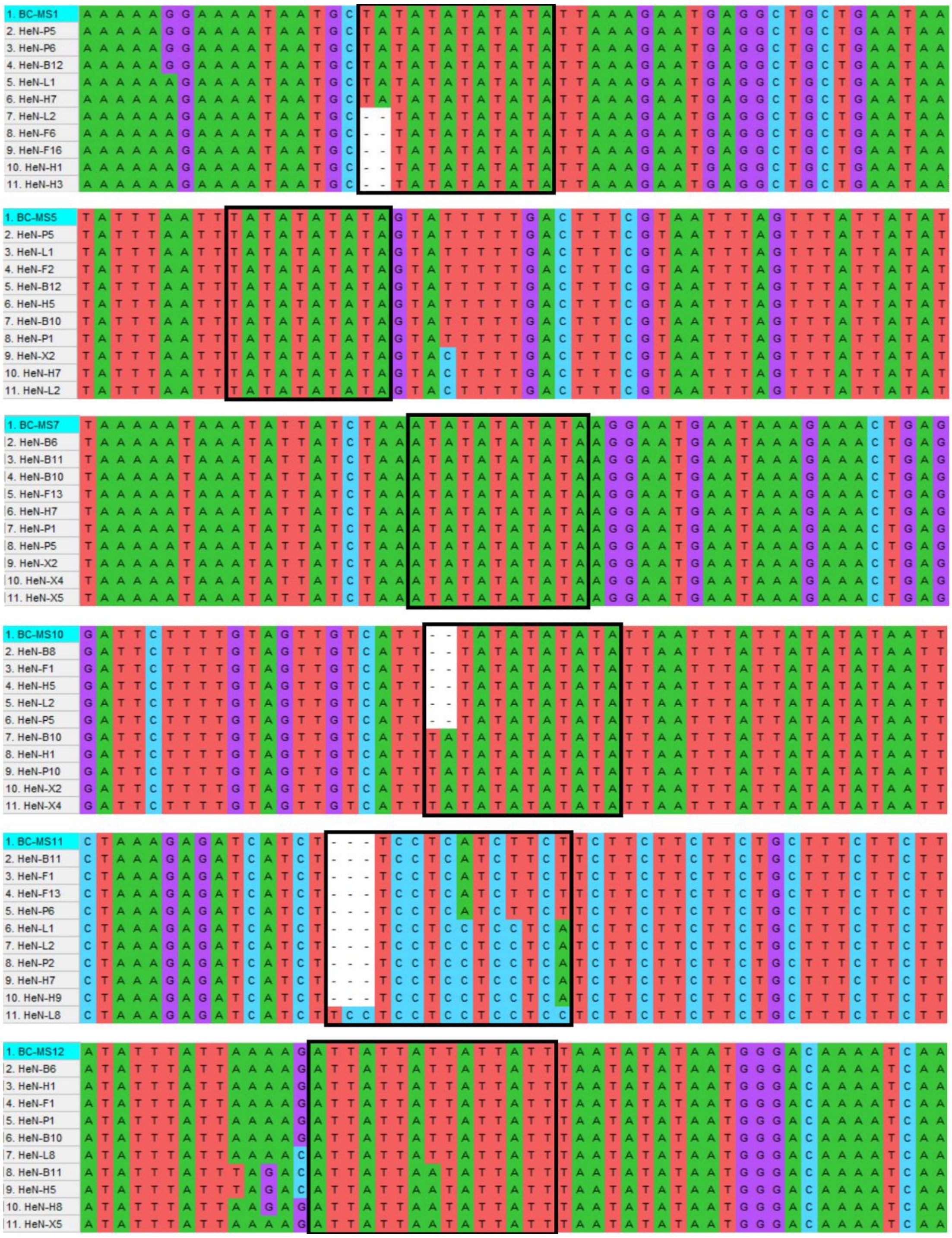

FIG S2. The sequences marked with black boxes are the core regions of microsatellites at the five loci (BC-MS1, BC-MS5, BC-MS10, BC-MS11, BC-MS12).

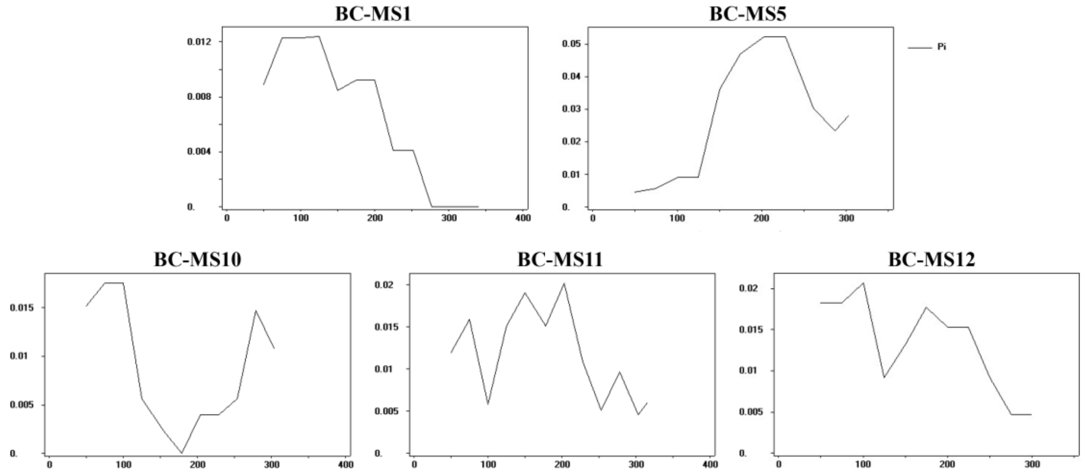

FIG S3. The nucleotide diversity of BC-MS1, BC-MS5, BC-MS10, BC-11and BC-MS12 loci in *B. coli*. X axis represents the nucleotide position, Y axis represents the value of nucleotide diversity (Pi).
